# Mentalized Affectivity, Helicopter Parenting, and Psychopathological Risk in Emerging Adults: A Network Analysis

**DOI:** 10.3390/ejihpe14090167

**Published:** 2024-09-18

**Authors:** Gianluca Cruciani, Andrea Fontana, Ilaria Maria Antonietta Benzi, Lucia Sideli, Laura Antonia Lucia Parolin, Laura Muzi, Nicola Carone

**Affiliations:** 1 Department of Systems Medicine, University of Rome Tor Vergata, Via Montpellier 1, 00133 Rome, Italy; gianluca.cruciani@uniroma2.it (G.C.); nicola.carone@uniroma2.it (N.C.); 2 Department of Human Science, LUMSA University, Piazza delle Vaschette 101, 00193 Rome, Italy; a.fontana2@lumsa.it (A.F.); l.sideli@lumsa.it (L.S.); 3 Department of Brain and Behavioral Sciences, University of Pavia, Piazza Botta 11, 27100 Pavia, Italy; imabenzi@gmail.com; 4 Department of Psychology, University of Milan—Bicocca, Piazza dell’Ateneo Nuovo 1, 20126 Milan, Italy; laura.parolin@unimib.it; 5 Department of Philosophy, Social Sciences, Humanities and Education, University of Perugia, Piazza G. Ermini 1, 06123 Perugia, Italy

**Keywords:** mentalized affectivity, helicopter parenting, depression, anxiety, somatization, emerging adulthood

## Abstract

Emerging adulthood represents a critical stage characterized by heightened risks for anxiety, depression, and somatization symptoms development. Research has shown that difficulties in emotional identification, expression, and processing, as well as dysfunctional parenting styles, may exacerbate symptoms in emerging adults. The present study aimed at examining the interplay between mentalized affectivity (i.e., emotional identification, processing, and expression), helicopter parenting, and psychopathological risk in 913 Italian cisgender emerging adults (*M* = 24.34, *SD* = 2.81; 71.20% assigned female at birth), using network analysis. The results indicated moderate to strong associations between psychopathological symptoms, with emotional processing difficulties significantly associated with general anxiety, depression, and, to a lesser extent, somatization. Additionally, increased degrees of helicopter parenting from mothers were linked to increased psychopathology and higher emotional processing difficulties. These findings emphasize the importance of addressing the interconnection between symptoms and emotional processing to prevent and treat psychopathological risks in emerging adults. Moreover, interventions targeting intrusive and overprotecting parenting behaviors may promote well-being among emerging adults.

## 1. Introduction

Emerging adulthood (18–29 years old) [1,2] is associated with significant contextual and social role changes. These changes may affect several life domains, including identity (through the exploration of diverse worldviews), stability (due to multiple life transitions), self-focus (associated with decreasing social obligations and responsibilities toward others), and future potential and possibilities [3]. As emerging adults navigate these changes, they may experience a lack of clear direction [4]. Moreover, socioeconomic factors (e.g., financial instability, access to higher education, and employment opportunities) may create inequalities, making it difficult for some to achieve their aspirations and establish a stable adult life [5,6].

Given the extent of change during this period, research on emerging adulthood has underlined a critical need to investigate psychological health and age-specific risk factors for psychopathology (e.g., [7,8,9,10]). While a significant percentage of emerging adults seem able to thrive on the opportunities presented during this time, others become bewildered and develop severe mental health issues, characterized by increased psychological distress, with a proportion of 62.5% of individuals with the onset of any mental disorders before the age of 25 [11,12,13,14]. Notably, the late teens and 20s are the typical period of onset for several mental disorders, including eating, personality, and psychotic disorders [14]. Increased substance use and externalizing symptoms have also been reported in this age range [15].

Relative to other mental health issues, anxiety disorders are the most prevalent among emerging adults. According to the National Comorbidity Survey Replication (NCS-R), approximately one-quarter of emerging adults in the United States meet the diagnostic criteria for an anxiety disorder, with specific phobia (10.3%), social anxiety disorder (9.1%), adult separation anxiety disorder (4.0%), panic disorder (2.8%), generalized anxiety disorder (2.0%), and agoraphobia (1.0%) most common [16]. High rates of anxiety disorders have also been reported among college students, showing a prevalence of 16.7% regarding generalized anxiety disorder and 4.5% concerning panic disorder in the previous year [17]. Anxiety disorders in emerging adults are more frequent among individuals assigned female at birth [18] and are associated with significant impairment, worsened psychosocial functioning, increased risk for suicidal thoughts or attempts, and adverse long-term developmental consequences [19].

Similarly, increasing rates of depressive disorders have been observed within this developmental stage. In more detail, national surveys have indicated that emerging adulthood is a period during which most individuals with a major depressive disorder report its onset (e.g., [20]). Furthermore, the 2021 National Survey on Drug Use and Health [21] reported that the prevalence of major depressive episodes was highest among individuals aged 18–25 years old, with 18.6% affected. Even higher rates have been observed among university students in low- and middle-income countries, with 24.4% experiencing depressive symptoms [22]. Assigned females have been found to be more likely to report depressive symptoms in emerging adulthood, possibly due to differences in childhood adversity or biological stress reactivity [23]. Furthermore, significantly higher mood disorder indicators, suicide-related outcomes, and rates of death by suicide have been observed among individuals aged 18–25 years old [24].

Notably, psychological distress (in terms of anxiety and depressive symptoms) among emerging adults has been associated with persistent somatic symptoms (e.g., musculoskeletal pain, headache, gastrointestinal dysfunction) and functional somatic syndromes (e.g., irritable bowel syndrome) [25]. Emerging adults have also been found to be more prone to somatoform disorders and functional somatic syndromes than individuals in other age groups [26]. A European survey indicated that 9.1–23.5% of college students meet the criteria for somatoform syndrome, characterized by disturbing physical symptoms with no known cause [25,27]. Additionally, studies have found that 10–20% of college students are diagnosed with a functional somatic disorder [28,29].

### 1.1. Mentalized Affectivity in Emerging Adulthood

Mentalized affectivity considers the interaction between emotion regulation and mentalizing processes in the interpretation of one’s own and other people’s actions and mental states, including their aspirations, feelings, and beliefs [30,31,32]. While emotion regulation refers to physiological, behavioral, and cognitive processes that enable individuals to modulate the experience and expression of positive and negative emotions, mentalization is defined as the “mental process by which an individual implicitly and explicitly interprets the actions of oneself and others as meaningful on the basis of intentional mental states such as personal desires, needs, feelings, beliefs, and reasons” [30]. Within this framework, mentalized affectivity can be conceptualized as the ability to reflect on one’s own emotions in light of personal experiences, cognitive processes, values, and personality [31]. The concept of mentalized affectivity encompasses three aspects of emotion regulation: identification (i.e., recognizing and reflecting on emotions and their influences), processing (i.e., modulating and distinguishing complex emotions), and expression (i.e., expressing emotions outwardly or inwardly) [31]. Effective emotion regulation requires mentalization, which is the capacity to contemplate one’s thoughts and feelings along with mentally prepare for future events that may have an emotional impact [33].

The process of identifying, expressing, and regulating emotions appears to increase in complexity during emerging adulthood [34]. More specifically, mentalized affectivity requires the ability to recognize the link between the emotional experience and the context, to individuate the origin of the emotion, and to integrate emotional and cognitive skills, all operations involving higher-order cognitive activity that keep maturing until emerging adulthood [35] (for a review, see [36]). It is likely that effective mentalized affectivity facilitates the formation of interpersonal bonds as well as intrapersonal connection to personal emotional experiences while also protecting against psychopathological symptoms [37,38].

In an effort to reexamine the significance of emotions in light of personal experiences and autobiographical memories, mentalized affectivity combines and integrates affect and cognition [33]. The ability to observe, be aware of, and reflect on emotions is the main focus of the mentalizing component of mentalized affectivity, which, in turn, refers to processes involved in reflective functioning about self and other, cognition and affect, based on internal and external features, including empathy, mindfulness, theory of mind, psychological mindedness, alexithymia, and insightfulness [39]. Studies have shown impaired mentalization in depressed adults (e.g., [40]) and children (e.g., [41]) and less recognition and interpretation of mental states in anxious individuals [42]. Moreover, patients with somatic disorders have been shown to exhibit deficits in the identification of their own’s emotions [43] and in the mentalization of others’ mental states [44]. Notably, a few studies have found that individuals with anxiety and mood disorders tend to exhibit impaired emotional processing relative to healthy controls [33]. However, more research on the potential relationship between mentalized affectivity and psychopathological risk is needed, particularly in samples of emerging adults.

### 1.2. Emerging Adults and Helicopter Parenting

While much empirical research has examined the bidirectional influence between parenting styles and psychopathology in children and adolescents, less attention has been given to the effects of parenting on mental health outcomes in emerging adults. Helicopter parenting, characterized by excessive parental involvement and control over offspring’s lives, is particularly noteworthy to be investigated in emerging adulthood (for a review, see [45]). Helicopter parents tend to “hover” over their offspring, frequently intervening to prevent failure, solve problems, and ensure success. This approach involves high levels of protection and supervision [46]. Helicopter parenting has been reported across cultures and social classes [47], and it has been found to be associated with increased rates of anxiety and depression in children and adolescents [45,48].

Research has also suggested that helicopter parenting may affect the development and well-being of emerging adults (e.g., [46,48,49,50]) when it is expected that they reach higher levels of autonomy and exploration, alongside greater independence in decision-making and finances, the adoption of adult obligations and duties, and dedication to steady love relationships, occupations, and residences [2]. Research on parent–child interaction during emerging adulthood suggests that a modification—but not elimination—of the parent–child relationship occurs during this period, underscoring the importance of appropriate parenting to foster healthy offspring adjustment (e.g., [51,52]). From the perspective of self-determination theory [53], helicopter parenting may interfere with emerging adults’ relatedness, competence, and autonomy needs—all critical psychological needs associated with well-being—and reduce social connections and interpersonal communication, resulting in lower self-efficacy [54,55].

Regarding psychopathological outcomes, emerging adults with helicopter parents have been found to exhibit heightened alcohol use (e.g., [56]), impaired self-regulation [57], dissatisfaction with psychological needs [54], and maladaptive perfectionism [58]. Research has also shown that helicopter parenting may lead to elevated depressive symptoms in the considered population due to a diminished self-perception of being autonomous and competent [48], deficits in self-control [59], an impaired sense of authenticity [60], and emotional dysregulation [61]. Similarly, helicopter parenting has been associated with increased generalized anxiety [59] and low physical self-esteem [62].

### 1.3. The Present Study

To the best of our knowledge, there is a lack of research assessing the interrelation between mentalized affectivity, current experiences of dysfunctional parenting, and psychopathological symptoms in emerging adults. Accordingly, the present study aimed at exploring psychopathological risk among a sample of cisgender emerging adults by investigating the associations between anxiety, depression, somatization, mentalized affectivity, and helicopter parenting using a network analysis approach. Network analysis represents a novel approach for conceptualizing psychopathology and related risk factors, presenting variables of interest as distinct nodes connected by edges indicating strength (e.g., strong/weak correlations) and direction (e.g., positive/negative correlations) [63].

In accordance with the literature, we hypothesized that anxiety, depression, and somatization symptoms would represent central nodes with strong interrelations. Furthermore, we hypothesized that psychopathological symptoms would be associated with significant impairment in mentalized affectivity, particularly in the realm of emotional processing, and that there would be a significant association between psychopathological symptomatology (especially depression and anxiety symptoms) and helicopter parenting.

## 2. Materials and Methods

### 2.1. Participants

The study utilized a non-probability cross-sectional community sample of 962 cisgender emerging adults aged 18–29 years old (*M* = 24.32, *SD* = 2.80). Among them, 687 (71.41%) were assigned female at birth. With respect to sexual identity, 74.01% (*n* = 712) identified as heterosexual, 11.12% (*n* = 107) as lesbian/gay, and 14.87% (*n* = 143) as bisexual+. The whole sample was a native Italian speaker who lived in Italy; almost the whole sample (*n* = 925, 96.15%) had Italian citizenship. The largest portion (*n* = 573, 59.56%) comprised students, while 283 (29.42%) were employed, 48 (4.99%) were unemployed, and 58 (6.03%) did not report their employment status. Most resided with their parents (*n* = 697, 72.45%), while 15.80% (*n* = 152) lived alone, 11.33% (*n* = 109) lived with a partner, and 0.42% (*n* = 4) cohabited with their child (ren).

### 2.2. Procedure

Participants were recruited using a referral sampling strategy. Participation in the study required acknowledgment and acceptance of an informed consent form presented to participants before they began the survey on the Qualtrics platform. Completing the survey was estimated to take approximately 20 min. The survey was filled in using a personal device not in the research lab and was designed to prevent the identification of individual respondents; therefore, responses were aggregated to ensure anonymity. The Ethics Committee of the University of Milan–Bicocca and the Territorial Ethics Committee Lazio Area 2 approved the research.

### 2.3. Measures

Psychopathological risk was operationalized into three aspects: anxiety, depression, and somatization.

**Anxiety**. Anxiety severity over the past 14 days was assessed using the Generalized Anxiety Disorder-7 (GAD-7; [64]) scale, which consists of seven items (e.g., “Feeling nervous, anxious, or on edge”) rated on a four-point Likert scale ranging from 0 (*not at all*) to 3 (*nearly every day*). Total scores range from 0–21, with higher scores indicating greater anxiety severity. Cronbach’s alpha in the present study was 0.88.

**Depression**. Depression severity was assessed using the Patient Health Questionnaire-9 (PHQ-9; [65]), which consists of nine DSM-IV depression criteria [65,66] (e.g., “Little interest or pleasure in doing things”). Each item is rated on a scale ranging from 0 (*not at all*) to 3 (*nearly every day*), reflecting the frequency of symptoms over the past 14 days. Cronbach’s alpha in the present study was 0.85.

**Somatization**. Somatization severity was assessed using the Patient Health Questionnaire-15 [67], which includes the most prevalent DSM-IV somatization disorder symptoms. The measure includes 13 symptom-related items that are rated with respect to their severity over the past 14 days on a range varying between 0 (*not bothered at all*) and 2 (*bothered a lot*); additionally, the questionnaire includes two items assessing physical symptoms (i.e., feeling tired or having little energy, trouble sleeping), on a scale with 0 (*not at all*), 1 (*several days*), or 2 (*more than half the days* or *nearly every day*). PHQ-15 total scores range between 0 and 30. Cronbach’s alpha in the present study was 0.79.

**Helicopter Parenting**. Emerging adults’ current perceptions of helicopter parenting (e.g., “My parent supervised my every move growing up”) were assessed using the 15-item Helicopter Parenting Instrument (HPI; [68,69]). HPI items are rated on a Likert scale with a range varying between 1 (*completely disagree*) and 7 (*completely agree*); higher ratings correspond to greater helicopter parenting experiences. Participants in the current study rated their mother’s and father’s helicopter parenting separately, completing the questionnaire twice. Cronbach’s alphas in the present study were 0.80 for maternal helicopter parenting and 0.78 for paternal helicopter parenting.

**Mentalized Affectivity**. The three core components of mentalized affectivity—emotional identification, processing, and expression—were assessed using the 12-item Brief-Mentalized Affectivity Scale (B-MAS; [32,70]) on a Likert scale with 7 points varying between 1 (*disagree strongly*) and 7 (*agree strongly*). Emotional identification involves recognizing and labeling one’s feelings and understanding their origins (e.g., “I try to put effort into identifying my emotions”); emotional processing pertains to the use of implicit strategies to regulate emotional intensity and duration (e.g., “It is hard for me to manage my emotions”); and emotional expression refers to the effective communication of feelings to others (e.g., “I often keep my emotions inside”). Cronbach’s alphas in the present study were 0.78 for identification, 0.81 for processing, and 0.82 for expression.

### 2.4. Data Analytic Plan

Data analyses were conducted using R statistical software version 4.3.3 [71] and the relevant packages for network analysis estimation and visualization (see Supplementary Materials for details). First, multivariate outliers were detected using Mahalanobis distance and excluded from the sample [72]. Subsequently, normality assumptions were assessed, and Pearson correlations were calculated to evaluate the associations between variables.

Second, bootstrap procedures were employed to assess the stability and consistency of both centrality and bridge indices. Analyses for the calculation of stability coefficients were run for strength, bridge strength, and indices of expected influence centrality [73]. Correlation networks were then constructed using the graphical least absolute shrinkage and selection operator (GLASSO) algorithm [74,75].

Following this, centrality indices, including expected influence, strength, closeness, and betweenness, were computed to identify key nodes. Both expected influence and strength measure the total of a node’s edge weights; absolute values were employed for strength [76,77]. Closeness centrality measures a node’s proximity to others by the reciprocal of the sum of shortest path distances, indicating nodes that can efficiently disseminate information [72,77]. Betweenness centrality quantifies the frequency with which a node acts as a bridge on the shortest path between other nodes, indicating a significant influence on information flow [73,78]. Bridge centrality analysis was performed to identify nodes connecting distinct psychological constructs within the network [79]. Finally, network group invariance was assessed using the network comparison test (NCT) to determine the network structure consistency across assigned sex at birth, ensuring equivalence between males and females [80].

## 3. Results

### 3.1. Missing Values Analysis and Descriptive Statistics

The dataset showed no missing values. Forty-nine participants (5.1% of the sample), including 12 assigned males at birth (24% of the outliers), were identified as multivariate outliers using Mahalanobis distance with *p* < 0.001. Following the removal of these outliers, the final sample consisted of 913 emerging adults (71.20% assigned female at birth, *n* = 650; 74.59% heterosexual, *n* = 681; 14.57% bisexual+, *n* = 133; 10.84% lesbian/gay, *n* = 99), aged 18–29 years old (*M* = 24.34, *SD* = 2.81). All variables were normally distributed (see Table 1).

### 3.2. Associations between Dimensions

**Figure 1:**
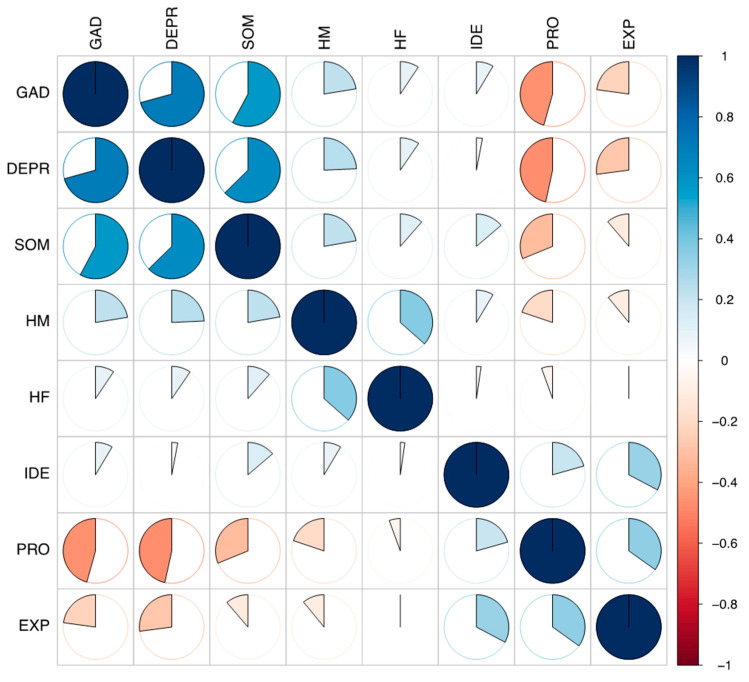
Zero-order correlations among the study’s variables (N = 913). *Note*. DEPR = depressive symptoms; GAD = anxiety; SOM = somatization; HF = paternal helicopter parenting; HM = maternal helicopter parenting; IDE = identifying emotions; PRO = processing emotions; EXP = expressing emotions.

As shown in Figure 1, psychopathological symptoms exhibited moderate to strong associations. Emotional processing difficulties were significantly linked to anxiety and depression, while emotional identification difficulties correlated with higher somatization. Maternal and paternal helicopter parenting were correlated; however, an increase in depressive and somatization symptomatology and greater emotional processing difficulties were correlated with maternal helicopter parenting only.

### 3.3. Network Stability

Bootstrap analyses confirmed the network accuracy (see Supplementary Figures S1 and S2), demonstrating comparable stability for both strength and expected influence indices (see Supplementary Figure S3). These analyses validated the significant impact of psychopathology, helicopter parenting, and mentalized affectivity on network activation [81,82]. All indexes had correlation stability coefficients of 0.75, suggesting that up to 75% of all cases could be removed while maintaining a 95% probability of preserving a 0.7 correlation [73]. Thus, indices of centrality and bridge were interpretable, offering insights into the roles and influences of different nodes and edges.

### 3.4. Network Estimation

**Figure 2:**
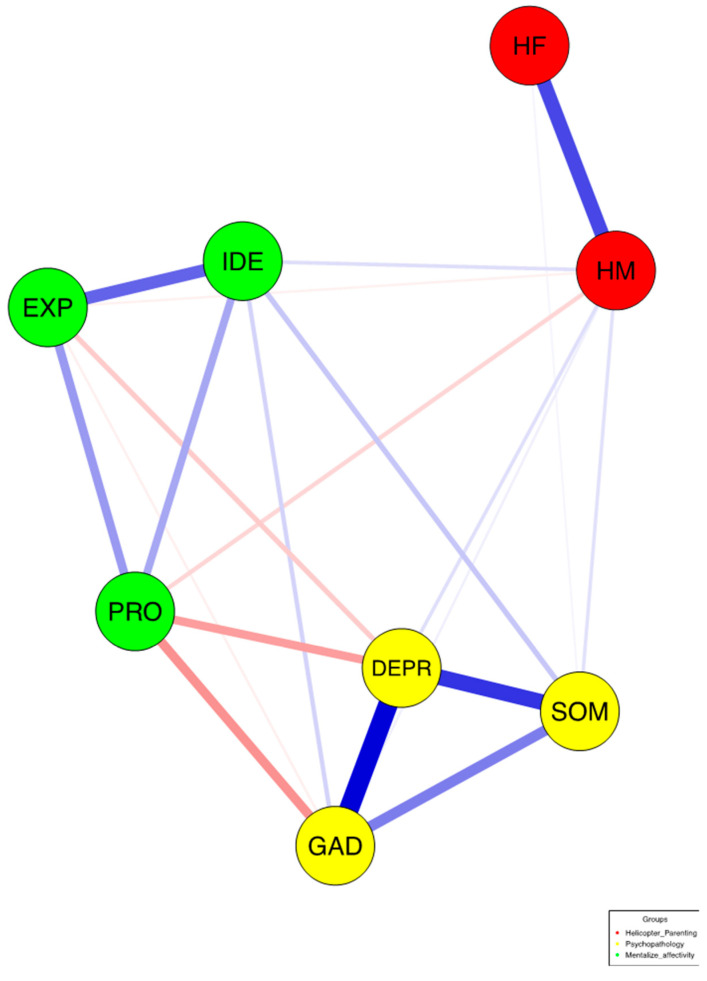
EBICglasso network depicting mentalized affectivity, helicopter parenting, and psychopathological scales in emerging adults (N = 913). *Note*. Nodes represent variables, and edges depict the relation between two variables, controlling for all other variables. Thicker edges indicate stronger positive partial correlations. DEPR = depressive symptoms; GAD = anxiety; SOM = somatization; HF = paternal helicopter parenting; HM = maternal helicopter parenting; IDE = identifying emotions; PRO = processing emotions; EXP = expressing emotions.

The network comprised 8 nodes and 20 edges out of 28 non-zero-order correlations, with a sparsity of 0.286 and a mean weight of 0.063 (Figure 2). This suggests that 71.4% of possible edges were present, indicating a moderate level of connectivity among nodes.

### 3.5. Network Inference

**Figure 3:**
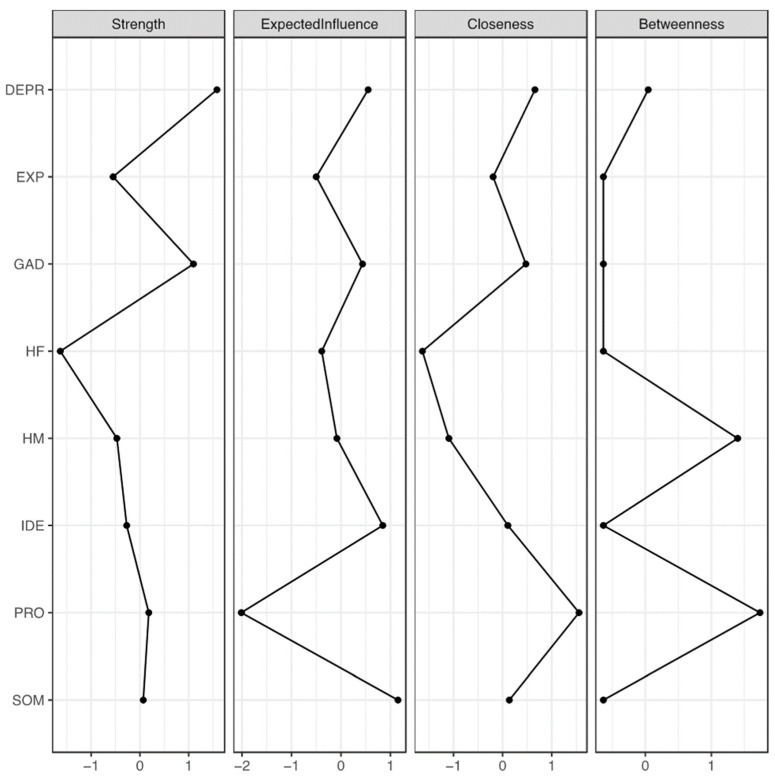
Centrality indices of the network variables (N = 913). *Note.* Centrality indices (i.e., node strength, expected influence, closeness, and betweenness) are shown as standardized z-scores. DEPR = depressive symptoms; GAD = anxiety; SOM = somatization; HF = paternal helicopter parenting; HM = maternal helicopter parenting; IDE = identifying emotions; PRO = processing emotions; EXP = expressing emotions.

The centrality indices showed that depression had the highest strength and expected influence, making it a pivotal node with strong connections and significant network impact (see Figure 3 and Table 2). Anxiety and somatization followed closely, highlighting their critical roles. Emotional processing had the greatest closeness and betweenness, acting as a key connector despite its negative expected influence and bridge strength on psychopathology nodes (see Supplementary Figures S4–S6). Maternal helicopter parenting had higher betweenness and closeness than paternal helicopter parenting, indicating a more central role in influencing psychopathology and emotional processing.

**Figure 4:**
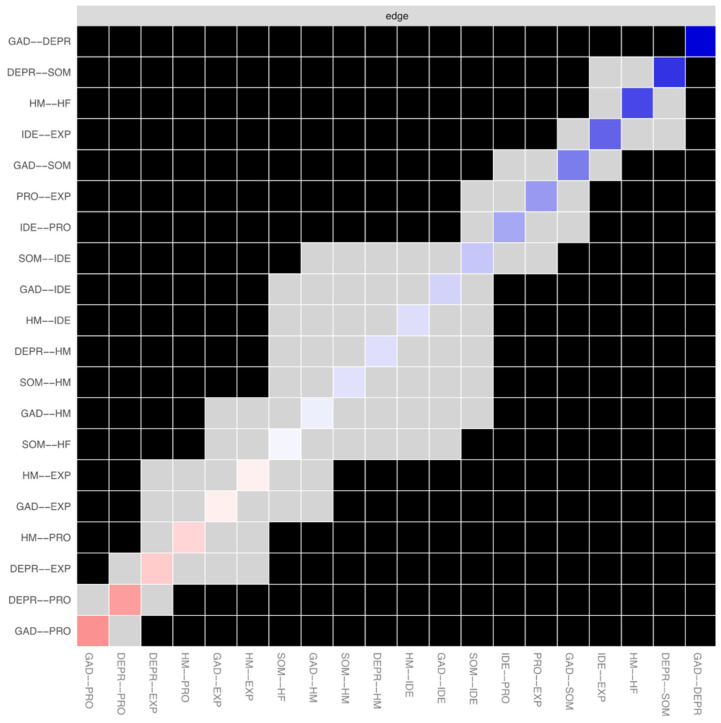
Bootstrapped edge-weights difference test of the network variables (N = 913). *Note*. Diagonal indicates the edge colors with the corresponding direction and magnitude of the associations. Gray-colored boxes represent non-significant differences between two nodes, and black boxes represent significant differences between two nodes (*p* < 0.05). DEPR = depressive symptoms; GAD = anxiety; SOM = somatization; HF = paternal helicopter parenting; HM = maternal helicopter parenting; IDE = identifying emotions; PRO = processing emotions; EXP = expressing emotions.

Table 3 shows that all psychopathology dimensions were highly interrelated. Depression and anxiety had a strong positive correlation, reinforcing their centrality. Maternal and paternal helicopter parenting were also positively correlated. Emotional processing negatively correlated with depression and anxiety, while emotional identification positively correlated with somatization, linking emotion regulation difficulties with these symptoms. Figure 4 highlights the significant negative associations between emotion regulation and psychopathology, and between emotional processing and maternal helicopter parenting. The network analysis identified depression and anxiety as central nodes with high strength and expected influence, emphasizing their importance. Emotional processing served as a key connector with high closeness and betweenness, despite its negative expected influence. Maternal helicopter parenting, with significant betweenness and closeness, played an influential role, linked closely to psychopathological risk.

The range of node predictability varied between 0.13 for paternal helicopter parenting and 0.60 for depression (*M* = 0.336, *SD* = 0.167). A total of 34% of node variance was explained by connected nodes (see Supplementary Figure S7).

### 3.6. Gender Invariance

The network invariance test demonstrated gender invariance between assigned males and assigned females (*M* = 0.19, *p* = 0.07). The networks slightly differed in global strength, with higher strength observed for assigned females (*S* = 0.99, *p* < 0.03). Specifically, assigned females exhibited a significantly stronger association between the capacity to identify emotions and the ability to process emotions compared to assigned males (*E* = 0.19, *p* < 0.03).

## 4. Discussion

The present study explored key risk factors for psychopathological symptoms among cisgender emerging adults by assessing the relationships between anxiety, depression, somatization, mentalized affectivity, and current experiences of helicopter parenting using a network analysis approach. The results largely supported our hypotheses. Psychopathological symptoms (related to anxiety, depression, and somatization) were moderately to strongly associated. Moreover, difficulties in mentalized affectivity—particularly in emotional processing—were significantly linked to general anxiety and depression, and impaired emotional identification was associated with somatization. Additionally, maternal helicopter parenting correlated with higher psychopathological symptomatology (especially anxiety and depressive symptoms) and reduced emotional processing.

The results identified anxiety and depression as central nodes in the network analysis, highlighting their significant influence during emerging adulthood. The clinical and empirical literature indicates that high levels of anxiety and depression are linked to poor health and negative psychological outcomes in this population. Indeed, anxiety may hinder the successful transition into adulthood, limiting social interactions and worsening mood [83,84]. Additionally, severe anxiety symptoms have been shown to be associated with low academic self-efficacy [85] and increased suicidal behaviors [86]. High levels of depressive symptoms in emerging adults have been linked to enhanced burnout, lower salaries, and dysfunctional coping strategies [87]. Moreover, individuals who have experienced recurrent depressive episodes prior to the age of 20 years old stand at higher risk of developing severe and chronic symptoms, frequent suicidality, comorbid anxiety disorders, and poor psychosocial functioning [88].

Clinical implications may be drawn from these findings. First, early detection appears vital to reducing anxiety and depressive symptoms and disorders among emerging adults. In this vein, several interventions targeting individuals aged 16 years old and older [89,90] have indicated that early detection is associated with better access to psychiatric care and lower use of emergency departments. Second, tailored prevention strategies and psychosocial interventions targeting these symptoms could significantly improve overall mental health in this population by identifying an individual’s unique symptom profile and providing information that is likely to be helpful based on said profile. Several programs that considered specific features of targeted individuals (including specific symptoms, distorted cognitive processes or biases, dysfunctional beliefs, and interpersonal patterns) have shown their effectiveness in contrasting mood and anxiety disorders among emerging adults (e.g., [91,92,93]). Lastly, it seems important to establish pertinent factors involved in psychopathological symptomatology during such a developmental period, with particular regard to their development and maintenance. Demographic variables, including sex assigned at birth, low family socioeconomic status, low parental education, as well as more specific factors such as student’s academic performance, learning disabilities, and externalizing symptoms, have been largely identified as risk factors for anxiety and depression among emerging adults (see, for example, [94]). In the present study, two other additional areas for potential risk factors were investigated, namely emerging adults’ mentalized affectivity and current experiences of helicopter parenting, which are discussed in the following paragraphs.

The individual variable of mentalized affectivity emerged as significantly associated with psychopathological risk. Specifically, the ability to process emotions was linked to anxiety and depressive symptoms. Additionally, decreased emotional identification skills were associated with greater somatization symptoms. Research has consistently shown that emotion regulation strategies play a key role in emerging adults’ mental health (e.g., [34,61]). Studies have further suggested that emerging adults engage in different emotion regulation patterns compared to individuals in other developmental stages, underlining the uniqueness of this developmental period. More specifically, it has been suggested that emotion regulation in older individuals is more selective and effective: in contrast to emerging adults, individuals in their middle adulthood are characterized by more adaptive emotion regulation strategies, but they are also marked by greater avoidance of feelings of anger, greater apathy toward sadness, and lower levels of search for social support when experiencing sadness or anger (e.g., [95]). This matches the aforementioned data on mentalized affectivity, showing that the identifying and processing emotions components correlate positively with age [35]. In other words, it is possible to speculate that emerging adults are still characterized by not-fully developed emotion regulation skills that should be targeted when planning interventions for this population.

Notably, difficulties in emotion regulation have been linked to several psychopathological conditions, including depression [96], anxiety [97], feeding and eating disorders [98], borderline personality disorder [99], and substance and behavioral addictions [100]. Similar results were also confirmed among emerging adults, whose maladaptive emotion regulation strategies have been found to be associated with regular and heavy cannabis use [101], problematic gambling, mood disorders [102], and social anxiety [103].

The study findings also align with the only previous study to have tested the associations between mentalized affectivity and psychopathological symptoms, which found a correlation between emotional processing difficulties and enhanced anxious symptomatology and mood disorders [33]. Furthermore, difficulties identifying and describing feelings, as well as differentiating somatic sensations and feelings (both crucial components of alexithymia), have been observed in emerging adults [104].

Clinically, the significant associations between emotional processing and identifying difficulties and psychopathological risk suggest that enhanced emotion regulation may disrupt the association with and between nodes representing psychopathological symptoms within this network. Indeed, interventions targeting emotional processing have been shown to reduce psychopathological symptoms among emerging adults (for a meta-analysis, see [105]). For example, Gatto et al. [106] developed an online brief emotion regulation training program for emerging adults aged between 18 and 23 years old, demonstrating that, after 5 weeks, improved emotion regulation abilities led to decreased psychological distress, in terms of depressive and anxiety symptoms, interpersonal functioning, and social role functioning. Moreover, gender-specific interventions may be necessary to address the differing strengths and associations between emotional identification and processing between assigned males and females.

In this vein, the present findings demonstrated gender invariance, with individuals assigned female at birth exhibiting a significantly stronger association between emotional identification and emotional processing. Research has shown that individuals assigned female at birth typically demonstrate superior emotional identification relative to individuals assigned male at birth [107,108]. In turn, higher emotional identification has been shown to correlate with better emotion regulation and processing [109,110]. Also, recent findings have confirmed this trend [32,70]. Future interventions should therefore aim at enhancing emotional identification to improve emerging adults’ capacities to process and regulate emotions while tailoring this training to accommodate gender differences. As aforementioned, taking into consideration specific strengths and weaknesses could be of help in the construction of effective programs that could take advantage of processing abilities among individuals assigned male at birth and of emotional identification among individuals assigned female at birth for enhancing emotion regulation skills.

A further variable of interest in the network analysis was helicopter parenting. In particular, maternal helicopter parenting was associated with greater psychopathological symptomatology and impaired emotional processing. This aligns with previous research showing that current experiences of dysfunctional parenting may negatively affect emerging adults’ well-being. In more detail, McKinney et al. [111] showed that ineffective parenting—characterized by higher levels of authoritarian and permissive styles, conflict, and harsh discipline—correlated with poorer psychological adjustment (i.e., increased internalizing and externalizing problems) in emerging adult offspring. Moreover, helicopter parenting has been associated with negative mental health outcomes in emerging adults, including elevated anxiety and depressive symptoms and emotion regulation difficulties [48,54,112].

In the current study, maternal helicopter parenting was strongly associated with psychopathological risk in emerging adults, and the network analysis revealed a significant link between helicopter parenting from mothers and fathers. In this vein, maladaptive parenting by both parents constitutes a significant risk factor for the development of psychopathological symptomatology in emerging adults. Such evidence supports the development of interventions aimed at promoting effective parenting modalities (e.g., non-intrusive problem-solving and support, promotion of children’s autonomy), which may mitigate psychopathological symptoms and improve emotional processing in emerging adults. Additionally, the literature suggests that the relationship between parenting styles and child psychological adjustment may be influenced by the offspring’s developmental stage. Despite beliefs suggesting that parental impacts diminish as children get older, there is growing evidence that parents still have a considerable influence on how well their offspring fare. Rather, meta-analytic studies have shown an enhancement in the relationship between internalizing and externalizing symptoms in children and parenting styles as age increases [113,114]. To the extent that specific literature on how helicopter parenting may be associated with individuals’ adjustment at different developmental stages is lacking, future longitudinal studies assessing this issue are recommended.

Evidence-based programs to improve parenting styles exist [115]. Yet, there is a pressing need to adapt these programs for parents of emerging adults—a need that is underscored by evidence showing that both emerging adults and their parents define adulthood using criteria such as autonomy and independence (e.g., accepting responsibility for one’s actions, making personal decisions) [51]. Future parenting programs should, therefore, address potential conflicts and expectations regarding autonomy and independence between emerging adults and their (helicopter) parents. Similarly, interventions with emerging adults should focus on helping them develop autonomy and resilience, which may have been hindered by helicopter parenting. To achieve this, emerging adults should be supported in becoming aware of how parenting patterns have impacted their current unmet psychological needs [53], as well as to understand that their own decisions and actions could trigger parents for what they do [52] and consequently mentalize upon the reasons behind parents’ behavior.

### Limitations

The present study has several limitations. First, participants were recruited using referral and volunteer sampling. Thus, they may not represent the broader Italian population of cisgender emerging adults. Additionally, it is possible that emerging adults with more severe psychopathological symptoms did not feel comfortable participating or encountered barriers to participation. Second, all measures were based on self-report questionnaires, which may have been susceptible to self-presentation and social desirability biases. Future studies should incorporate multi-informant assessments to overcome this issue. Third, most participants were heterosexual, with only one-fourth identifying as lesbian, gay, or bisexual+, thereby preventing exploration of whether the study variables were associated differently based on participants’ sexual orientation. Future studies should recruit a larger sample of individuals with minoritized sexual orientations to address this question. Fourth, the study employed a network analysis using a cross-sectional design, thereby limiting the ability to draw causal inferences. Longitudinal studies are recommended to explore the causal relationships between psychopathological risk, mentalized affectivity, and current parenting experiences among emerging adults. In the same vein, future studies are warranted to explore the role of different parenting styles, as helicopter parenting may be associated with different parenting profiles [116]. Fourth, as the GAD-7 is a measure of generalized anxiety disorder, conclusions regarding anxiety may be limited to this particular aspect of the anxiety spectrum.

## 5. Conclusions

Limitations notwithstanding, this is the first study that explored the relationship between emerging adults’ psychopathological symptoms (depression, anxiety, and somatization), mentalized affectivity, and current experiences of helicopter parenting using a network analysis approach. It provides valuable insights for targeting specific factors affecting mental health and psychopathological risk in emerging adulthood. Understanding the interconnected nature of these symptoms can help develop integrated and effective treatment programs to promote psychological well-being during this challenging developmental stage [117]. Future interventions involving emerging adults should consider the interconnection between anxiety, depression, and somatization, the role of mentalized affectivity (especially its emotional processing dimension), and the impact of helicopter parenting on the pursuit of independence and autonomy.

## Figures and Tables

**Table 1:** Descriptive statistics of the study variables (N = 913).

	*N*	*M*	*SD*	Skewness	Kurtosis
DEPR	913	0.954	0.577	0.795	0.244
EXP	913	3.641	1.323	0.103	−0.612
GAD	913	1.258	0.692	0.582	−0.481
HF	913	3.101	0.870	0.303	−0.227
HM	913	3.563	0.889	0.174	−0.313
IDE	913	5.084	1.180	−0.429	−0.190
PRO	913	4.066	1.208	−0.089	−0.091
SOM	913	0.548	0.314	0.563	−0.067

*Note*. DEPR = depressive symptoms; GAD = anxiety symptoms; SOM = somatization; HF = paternal helicopter parenting; HM = maternal helicopter parenting; IDE = identifying emotions; PRO = processing emotions; EXP = expressing emotions.

**Table 2:** Non-standardized centrality indexes for the network (N = 913).

	Centrality Indexes			
	Betweenness	Closeness	Strength	Expected Influence
GAD	0	0.014	1.011	0.563
DEPR	2	0.014	1.130	0.593
SOM	0	0.013	0.758	0.758
HM	6	0.009	0.624	0.421
HF	0	0.008	0.338	0.338
IDE	0	0.013	0.674	0.674
PRO	7	0.016	0.785	−0.105
EXP	0	0.012	0.605	0.307

*Note.* DEPR = depressive symptoms; GAD = general anxiety disorder symptoms; SOM = somatization; HF = paternal helicopter parenting; HM = maternal helicopter parenting; IDE = identifying emotions; PRO = processing emotions; EXP = expressing emotions.

**Table 3:** Edge weights between the nodes of the network (N = 913).

	GAD	DEPR	SOM	HM	HF	IDE	PRO	EXP
GAD	0.00	0.45	0.23	0.03	0.00	0.08	0.20	−0.03
DEPR	0.45	0.00	0.35	0.06	0.00	0.00	−0.17	−0.09
SOM	0.23	0.35	0.00	0.05	0.02	0.10	0.00	0.00
HM	0.03	0.06	0.05	0.00	0.32	0.06	−0.08	−0.03
HF	0.00	0.00	0.02	0.32	0.00	0.00	0.00	0.00
IDE	0.08	0.00	0.10	0.06	0.00	0.00	0.16	0.27
PRO	−0.20	−0.17	0.00	−0.08	0.00	0.16	0.00	0.18
EXP	−0.03	−0.09	0.00	−0.03	0.00	0.27	0.18	0.00

*Note.* Edge weights indicate regularized partial correlations between nodes. DEPR = depressive symptoms; GAD = general anxiety disorder symptoms; SOM = somatization; HF = paternal helicopter parenting; HM = maternal helicopter parenting; IDE = identifying emotions; PRO = processing emotions; EXP = expressing emotions.

## Data Availability

The data supporting the findings of this study are available from the corresponding author upon reasonable request.

## Supplementary Materials

The following supporting information can be downloaded at: https://www.mdpi.com/article/10.3390/ejihpe14090167/s1, Data analytic strategy for network estimation and assessment; Figure S1. Bootstrapped confidence intervals of estimated edge weights for the network (N = 913); Figure S2. Plotted case dropping for strength centrality index of the network (N = 913); Figure S3. Bootstrapped expected influence difference test of the network variables (N = 913); Figure S4. Bootstrapped strength centrality index difference test of the network variables (N = 913); Figure S5. Bridge centrality indices of the network variables (N = 913); Figure S6. Bootstrapped bridge strength difference test of the network variables (N = 913); Figure S7. Plot of node predictability depicting psychopathology, mentalized affectivity, and helicopter parenting scales in emerging adults (N = 913); Figure S8. Bootstrapped edge-weights difference test of the network variables (N = 913).

## References

[1] Arnett J.J. (2000). Emerging adulthood: A theory of development from the late teens through the twenties. Am. Psychol..

[2] Arnett J.J. (2023). Emerging Adulthood: The Winding Road from the Late Teens through the Twenties.

[3] Arnett J.J. (2007). Afterword: Aging out of care—Toward realizing the possibilities of emerging adulthood. New Dir. Youth Dev..

[4] Sumner R., Burrow A.L., Hill P.L. (2015). Identity and purpose as predictors of subjective well-being in emerging adulthood. Emerg. Adulthood.

[5] Grosemans I., Hannes K., Neyens J., Kyndt E. (2020). Emerging adults embarking on their careers: Job and identity explorations in the transition to work. Youth Soc..

[6] Wells R.S., Lynch C.M. (2012). Delayed college entry and the socioeconomic gap: Examining the roles of student plans, family income, parental education, and parental occupation. J. High. Educ..

[7] Benzi I.M.A., Carone N., Parolin L., Martin-Gagnon G., Ensink K., Fontana A. (2023). Different epistemic stances for different traumatic experiences: Implications for mentalization. Res. Psychother. Psychopathol. Process Outcome.

[8] Benzi IM A., Fontana A., Lingiardi V., Parolin L., Carone N. (2024). “Don’t leave me behind!” Problematic Internet use and fear of missing out through the lens of epistemic trust in emerging adulthood. Curr. Psychol..

[9] Carone N., Benzi I.M.A., Parolin L.A.L., Fontana A. (2023). “I can’t miss a thing”—The contribution of defense mechanisms, grandiose narcissism, and vulnerable narcissism to fear of missing out in emerging adulthood. Personal. Individ. Differ..

[10] Persike M., Seiffge-Krenke I., Cok F., Głogowska K., Pavlopoulos V., Tantaros S., Perchec C., Rohail I., Saravia J.C. (2020). Emerging adults’ psychopathology in seven countries: The impact of identity-related risk factors. Emerg. Adulthood.

[11] Arnett J.J. (2007). Emerging adulthood: What is it, and what is it good for?. Child Dev. Perspect..

[12] Reinherz H.Z., Paradis A.D., Giaconia R.M., Stashwick C.K., Fitzmaurice G. (2003). Childhood and adolescent predictors of major depressive disorder in the transition to adulthood. Am. J. Psychiatry.

[13] Schwartz S.J. (2016). Turning point for a turning point: Advancing emerging adulthood theory and research. Emerg. Adulthood.

[14] Solmi M., Radua J., Olivola M., Croce E., Soardo L., de Pablo G.S., Shin J.I., Kirkbride J.B., Jones P., Kim J.H. (2022). Age at onset of mental disorders worldwide: Large-scale meta-analysis of 192 epidemiological studies. Mol. Psychiatry.

[15] Stone A.L., Becker L.G., Huber A.M., Catalano R.F. (2012). Review of risk and protective factors of substance use and problem use in emerging adulthood. Addict. Behav..

[16] LeBlanc N.J., Brown M., Henin A., Bui E., Charney M., Baker A. (2020). Anxiety disorders in emerging adulthood. Clinical Handbook of Anxiety Disorders. Current Clinical Psychiatry.

[17] Auerbach R.P., Mortier P., Bruffaerts R., Alonso J., Benjet C., Cuijpers P., Demyttenaere K., Ebert D.D., Green J.G., Hasking P. (2018). WHO world mental health surveys international college student project: Prevalence and distribution of mental disorders. J. Abnorm. Psychol..

[18] Essau C.A., Lewinsohn P.M., Lim J.X., Ho M.R., Rohde P. (2018). Incidence, recurrence and comorbidity of anxiety disorders in four major developmental stages. J. Affect. Disord..

[19] Asselmann E., Beesdo-Baum K. (2015). Predictors of the course of anxiety disorders in adolescents and young adults. Curr. Psychiatry Rep..

[20] Kessler R.C., Berglund P., Demler O., Jin R., Merikangas K.R., Walters E.E. (2005). Lifetime prevalence and age-of-onset distributions of DSM-IV disorders in the National Comorbidity Survey Replication. Arch. Psychiatry.

[21] US Department of Health and Human Services (2022). Major Depression. https://www.nimh.nih.gov/health/statistics/major-depression#:~:text=Prevalence%20of%20Major%20Depressive%20Episode%20Among%20Adults,figure%201%20shows&text=The%20prevalence%20of%20major%20depressive,18%2D25%20(18.6%25).

[22] Akhtar P., Ma L., Waqas A., Naveed S., Li Y., Rahman A., Wang Y. (2020). Prevalence of depression among university students in low and middle income countries (LMICs): A systematic review and meta-analysis. J. Affect. Disord..

[23] Mondi C.F., Reynolds A.J., Ou S.R. (2017). Predictors of depressive symptoms in emerging adulthood in a low-income urban cohort. J. Appl. Dev. Psychol..

[24] Twenge J.M., Cooper A.B., Joiner T.E., Duffy M.E., Binau S.G. (2019). Age, period, and cohort trends in mood disorder indicators and suicide-related outcomes in a nationally representative dataset, 2005–2017. J. Abnorm. Psychol..

[25] Fischer S., Gaab J., Ehlert U., Nater U.M. (2013). Prevalence, overlap, and predictors of functional somatic syndromes in a student sample. Int. J. Behav. Med..

[26] Petersen M.W., Schröder A., Jørgensen T., Ørnbøl E., Dantoft T.M., Eliasen M., Carstensen T.W., Eplov L.F., Fink P. (2020). Prevalence of functional somatic syndromes and bodily distress syndrome in the Danish population: The DanFunD study. Scand. J. Public Health.

[27] Schlarb A.A., Claßen M., Hellmann S.M., Vögele C., Gulewitsch M.D. (2017). Sleep and somatic complaints in university students. J. Pain Res..

[28] Costanian C., Tamim H., Assaad S. (2015). Prevalence and factors associated with irritable bowel syndrome among university students in Lebanon: Findings from a cross-sectional study. World J. Gastroenterol..

[29] Gulewitsch M.D., Enck P., Hautzinger M., Schlarb A.A. (2011). Irritable bowel syndrome symptoms among German students: Prevalence, characteristics, and associations to somatic complaints, sleep, quality of life, and childhood abdominal pain. Eur. J. Gastroenterol. Hepatol..

[30] Bateman A.W., Fonagy P. (2004). Mentalization-based treatment of BPD. J. Personal. Disord..

[31] Jurist E. (2018). Minding Emotions: Cultivating Mentalization in Psychotherapy.

[32] Liotti M., Spitoni G.F., Lingiardi V., Marchetti A., Speranza A.M., Valle A., Jurist E., Giovanardi G. (2021). Mentalized affectivity in a nutshell: Validation of the Italian version of the Brief-Mentalized Affectivity Scale (B-MAS). PLoS ONE.

[33] Greenberg D.M., Kolasi J., Hegsted C.P., Berkowitz Y., Jurist E.L. (2017). Mentalized affectivity: A new model and assessment of emotion regulation. PLoS ONE.

[34] Charpentier-Mora S., Bastianoni C., Cavanna D., Bizzi F. (2024). Emerging adults facing the COVID-19 pandemic: Emotion dysregulation, mentalizing, and psychological symptoms. Curr. Psychol..

[35] Rinaldi T., Castelli I., Greco A., Greenberg D.M., Jurist E., Valle A., Marchetti A. (2021). The mentalized affectivity scale (MAS): Development and validation of the Italian version. PLoS ONE.

[36] Korzeniowski C., Ison M.S., Difabio de Anglat H., Gargiulo P.Á., Mesones Arroyo H.L. (2021). A summary of the developmental trajectory of executive functions from birth to adulthood. Psychiatry and Neuroscience Update.

[37] Brewer S.K., Zahniser E., Conley C.S. (2016). Longitudinal impacts of emotion regulation on emerging adults: Variable-and person-centered approaches. J. Appl. Dev. Psychol..

[38] Chan S., Rawana J.S. (2021). Examining the associations between interpersonal emotion regulation and psychosocial adjustment in emerging adulthood. Cogn. Ther. Res..

[39] Luyten P., Campbell C., Allison E., Fonagy P. (2020). The mentalizing approach to psychopathology: State of the art and future directions. Annu. Rev. Clin. Psychol..

[40] Ekeblad A., Falkenstrom F., Holmqvist R. (2016). Reflective functioning as predictor of working alliance and outcome in the treatment of depression. J. Consult. Clin. Psychol..

[41] Ensink K., Begin M., Normandin L., Fonagy P. (2016). Maternal and child reflective functioning in the context of child sexual abuse: Pathways to depression and externalising difficulties. Eur. J. Psychotraumatol..

[42] Chevalier V., Simard V., Achim J. (2023). Meta-analyses of the associations of mentalization and proxy variables with anxiety and internalizing problems. J. Anxiety Disord..

[43] Subic-Wrana C., Beutel M.E., Knebel A., Lane R.D. (2010). Theory of mind and emotional awareness deficits in patients with somatoform disorders. Psychosom. Med..

[44] Zunhammer M., Halski A., Eichhammer P., Busch V. (2015). Theory of mind and emotional awareness in chronic somatoform pain patients. PLoS ONE.

[45] Vigdal J.S., Brønnick K.K. (2022). A systematic review of “helicopter parenting” and its relationship with anxiety and depression. Front. Psychol..

[46] Segrin C., Woszidlo A., Givertz M., Bauer A., Murphy M.T. (2012). The association between overparenting, parent-child communication, and entitlement and adaptive traits in adult children. Fam. Relat..

[47] Ishizuka P. (2019). Social class, gender, and contemporary parenting standards in the United States: Evidence from a national survey experiment. Soc. Forces.

[48] Schiffrin H.H., Liss M., Miles-McLean H., Geary K.A., Erchull M.J., Tashner T. (2014). Helping or hovering? The effects of helicopter parenting on college students’ well-being. J. Child Fam. Stud..

[49] Carone N., Gartrell N.K., Rothblum E.D., Koh A.S., Bos H.M.W. (2022). Helicopter parenting, emotional avoidant coping, mental health, and homophobic stigmatization among emerging adult offspring of lesbian parents. J. Fam. Psychol..

[50] Carone N., Benzi I.M.A., Muzi L., Parolin L.A.L., Fontana A. (2023). Problematic Internet use in emerging adulthood to escape from maternal helicopter parenting: Defensive functioning as a mediating mechanism. Res. Psychother. Psychopathol. Process Outcome.

[51] Nelson L.J., Padilla-Walker L.M., Carroll J.S., Madsen S.D., Barry C.M., Badger S. (2007). “If you want me to treat you like an adult, start acting like one!” Comparing the criteria that emerging adults and their parents have for adulthood. J. Fam. Psychol..

[52] Nelson L.J., Padilla-Walker L.M., McLean R.D. (2021). Longitudinal predictors of helicopter parenting in emerging adulthood. Emerg. Adulthood.

[53] Deci E.L., Ryan R.M. (2008). Facilitating optimal motivation and psychological well-being across life’s domains. Can. Psychol..

[54] Cui M., Darling C.A., Coccia C., Fincham F.D., May R.W. (2019). Indulgent parenting, helicopter parenting, and well-being of parents and emerging adults. J. Child Fam. Stud..

[55] Reed K., Duncan J.M., Lucier-Greer M., Fixelle C., Ferraro A.J. (2016). Helicopter parenting and emerging adult self-efficacy: Implications for mental and physical health. J. Child Fam. Stud..

[56] Villegas-Pantoja M., Guzm’an-Facundo F., Alonso-Castillo M., de la Rubia J.M., López-Garcıa K. (2018). Parenting behaviors and their relationship with alcohol involvement in Mexican teenagers and young adults. J. Child Adolesc. Subst. Abus..

[57] Moilanen K.L., Lynn Manuel M. (2019). Helicopter parenting and adjustment outcomes in young adulthood: A consideration of the mediating roles of mastery and self-regulation. J. Child Fam. Stud..

[58] Hayes K.N., Turner L.A. (2021). The relation of helicopter parenting to maladaptive perfectionism in emerging adults. J. Fam. Issues.

[59] Hong P., Cui M. (2020). Helicopter parenting and college students’ psychological maladjustment: The role of self-control and living arrangement. J. Child Fam. Stud..

[60] Turner L.A., Faulk R.D., Garner T. (2020). Helicopter parenting, authenticity, and depressive symptoms: A mediation model. J. Genet. Psychol..

[61] Nguyen Q., Madison S., Ekas N.V., Kouros C.D. (2024). Helicopter parenting behaviors and emerging adult mental health: The mediating role of emotion dysregulation. Emerg. Adulthood.

[62] Wang C., Shi H., Li G. (2024). Helicopter parenting and college student depression: The mediating effect of physical self-esteem. Front. Psychiatry.

[63] Robinaugh D.J., Hoekstra R.H., Toner E.R., Borsboom D. (2020). The network approach to psychopathology: A review of the literature 2008–2018 and an agenda for future research. Psychol. Med..

[64] Spitzer R.L., Kroenke K., Williams J.B., Löwe B. (2006). A brief measure for assessing generalized anxiety disorder: The GAD-7. Arch. Intern. Med..

[65] Kroenke K., Spitzer R.L., Williams J.B. (2001). The PHQ-9: Validity of a brief depression severity measure. J. Gen. Intern. Med..

[66] Spitzer R.L., Kroenke K., Williams J.B.W., Patient Health Questionnaire Study Group (1999). Validity and utility of a self-report version of PRIME-MD: The PHQ Primary Care Study. JAMA.

[67] Kroenke K., Spitzer R.L., Williams J.B. (2002). The PHQ-15: Validity of a new measure for evaluating the severity of somatic symptoms. Psychosom. Med..

[68] Odenweller K.G., Booth-Butterfield M., Weber K. (2014). Investigating helicopter parenting, family environments, and relational outcomes for millennials. Commun. Stud..

[69] Pistella J., Izzo F., Isolani S., Ioverno S., Baiocco R. (2020). Helicopter mothers and helicopter fathers: Italian adaptation and validation of the Helicopter Parenting Instrument. Psychol. Hub.

[70] Greenberg D.M., Rudenstine S., Alaluf R., Jurist E.L. (2021). Development and validation of the Brief-Mentalized Affectivity Scale: Evidence from cross-sectional online data and an urban community-based mental health clinic. J. Clin. Psychol..

[71] R Core Team (2024). R: A Language and Environment for Statistical Computing.

[72] Tabachnick B.G., Fidell L.S. (2013). Using Multivariate Statistics.

[73] Epskamp S., Borsboom D., Fried E.I. (2018). Estimating psychological networks and their accuracy: A tutorial paper. Behav. Res. Methods.

[74] Foygel R., Drton M. Extended Bayesian information Criteria for Gaussian Graphical Models. Advances in Neural Information Processing Systems 23, Proceedings of the 24th Annual Conference on Neural Information Processing Systems, Vancouver, BC, Canada, 6–9 December 2010.

[75] Friedman J., Hastie T., Tibshirani R. (2008). Sparse inverse covariance estimation with the graphical lasso. Biostatistics.

[76] Bringmann L.F., Elmer T., Epskamp S., Krause R.W., Schoch D., Wichers M., Wigman J.T.W., Snippe E. (2019). What do centrality measures measure in psychological networks?. J. Abnorm. Psychol..

[77] Costantini G., Epskamp S., Borsboom D., Perugini M., Mõttus R., Waldorp L.J., Cramer A.O.J. (2015). State of the aRt personality research: A tutorial on network analysis of personality data in R. J. Res. Personal..

[78] Burger J., Isvoranu A.-M., Lunansky G., Haslbeck J.M.B., Epskamp S., Hoekstra R.H.A., Fried E.I., Borsboom D., Blanken T.F. (2023). Reporting standards for psychological network analyses in cross-sectional data. Psychol. Methods.

[79] Jones P.J., Ma R., McNally R.J. (2021). Bridge centrality: A network approach to understanding comorbidity. Multivar. Behav. Res..

[80] van Borkulo C.D., van Bork R., Boschloo L., Kossakowski J.J., Tio P., Schoevers R.A., Borsboom D., Waldorp L.J. (2023). Comparing network structures on three aspects: A permutation test. Psychol. Methods.

[81] Borsboom D. (2017). A network theory of mental disorders. World Psychiatry.

[82] Borsboom D., Cramer AO J. (2013). Network analysis: An integrative approach to the structure of psychopathology. Annu. Rev. Clin. Psychol..

[83] Hur J., DeYoung K.A., Islam S., Anderson A.S., Barstead M.G., Shackman A.J. (2020). Social context and the real-world consequences of social anxiety. Psychol. Med..

[84] Kranzler A., Elkins R.M., Albano A.M. (2019). Anxiety in emerging adulthood: A developmentally informed treatment model. Pediatric Anxiety Disorders.

[85] García A.G., Velazquez M.L. (2020). Relationship between academic self-efficacy, performance and anxious and depressive symptoms in emerging adult college students. Educación.

[86] Boden J.M., Fergusson D.M., Horwood L.J. (2007). Anxiety disorders and suicidal behaviours in adolescence and young adulthood: Findings from a longitudinal study. Psychol. Med..

[87] Salmela-Aro K., Aunola K., Nurmi J.E. (2008). Trajectories of depressive symptoms during emerging adulthood: Antecedents and consequences. Eur. J. Dev. Psychol..

[88] Rohde P., Lewinsohn P.M., Klein D.N., Seeley J.R., Gau J.M. (2013). Key characteristics of major depressive disorder occurring in childhood, adolescence, emerging adulthood, adulthood. Clin. Psychol. Sci..

[89] Anderson K.K., John-Baptiste A., MacDougall A.G., Li L., Kurdyak P., Osuch E.A. (2019). Access and health system impact of an early intervention treatment program for emerging adults with mood and anxiety disorders. Can. J. Psychiatry.

[90] Arcaro J., Summerhurst C., Vingilis E., Wammes M., Osuch E. (2017). Presenting concerns of emerging adults seeking treatment at an early intervention outpatient mood and anxiety program. Psychol. Health Med..

[91] Breedvelt J.J.F., Kandola A., Kousoulis A.A., Brouwer M.E., Karyotaki E., Bockting C.L.H., Cuijpers P. (2018). What are the effects of preventative interventions on major depressive disorder (MDD) in young adults? A systematic review and meta-analysis of randomized controlled trials. J. Affect. Disord..

[92] Hoffman L.J., Guerry J.D., Albano A.M. (2018). Launching anxious young adults: A specialized cognitive-behavioral intervention for transitional aged youth. Curr. Psychiatry Rep..

[93] Silfvernagel K., Wassermann C., Andersson G. (2017). Individually tailored internet-based cognitive behavioural therapy for young adults with anxiety disorders: A pilot effectiveness study. Internet Interv..

[94] LoParo D., Fonseca A.C., Matos AP M., Craighead W.E. (2024). Anxiety and depression from childhood to young adulthood: Trajectories and risk factors. Child Psychiatry Hum. Dev..

[95] Zimmermann P., Iwanski A. (2014). Emotion regulation from early adolescence to emerging adulthood and middle adulthood: Age differences, gender differences, and emotion-specific developmental variations. Int. J. Behav. Dev..

[96] Liu D.Y., Thompson R.J. (2017). Selection and implementation of emotion regulation strategies in major depressive disorder: An integrative review. Clin. Psychol. Rev..

[97] Cisler J.M., Olatunji B.O. (2012). Emotion regulation and anxiety disorders. Curr. Psychiatry Rep..

[98] Mirabella M., Carone N., Franco A., Rugo M.A., Speranza A.M., Mazzeschi C., Lingiardi V., Muzi L. (2024). Emotional dysregulation and eating symptoms in gender dysphoria and eating disorders: The mediating role of body uneasiness. Curr. Psychol..

[99] Daros A.R., Williams G.E. (2019). A meta-analysis and systematic review of emotion-regulation strategies in borderline personality disorder. Harv. Rev. Psychiatry.

[100] Estévez A., Jáuregui P., Sánchez-Marcos I., López-González H., Griffiths M.D. (2017). Attachment and emotion regulation in substance addictions and behavioral addictions. J. Behav. Addict..

[101] Nichols E.S., Penner J., Ford K.A., Wammes M., Neufeld RW J., Mitchell D.G.V., Greening S.G., Théberge J., Williamson P.C., Osuch E.A. (2021). Emotion regulation in emerging adults with major depressive disorder and frequent cannabis use. NeuroImage Clin..

[102] Marchica L.A., Keough M.T., Montreuil T.C., Derevensky J.L. (2020). Emotion regulation interacts with gambling motives to predict problem gambling among emerging adults. Addict. Behav..

[103] Jiao C., Cui M., Fincham F.D. (2024). Overparenting, loneliness, and social anxiety in emerging adulthood: The mediating role of emotion regulation. Emerg. Adulthood.

[104] Parolin M., Miscioscia M., De Carli P., Cristofalo P., Gatta M., Simonelli A. (2018). Alexithymia in young adults with substance use disorders: Critical issues about specificity and treatment predictivity. Front. Psychol..

[105] Moltrecht B., Deighton J., Patalay P., Edbrooke-Childs J. (2021). Effectiveness of current psychological interventions to improve emotion regulation in youth: A meta-analysis. Eur. Child Adolesc. Psychiatry.

[106] Gatto A.J., Elliott T.J., Briganti J.S., Stamper M.J., Porter N.D., Brown A.M., Harden S.M., Cooper L.D., Dunsmore J.C. (2022). Development and feasibility of an online brief emotion regulation training (BERT) program for emerging adults. Front. Public Health.

[107] Boden M.T., Thompson R.J., Dizén M., Berenbaum H., Baker J.P. (2013). Are emotional clarity and emotion differentiation related?. Cogn. Emot..

[108] Mankus A.M., Boden M.T., Thompson R.J. (2016). Sources of variation in emotional awareness: Age, gender, and socioeconomic status. Personal. Individ. Differ..

[109] Boden M.T., Thompson R.J. (2015). Facets of emotional awareness and associations with emotion regulation and depression. Emotion.

[110] Szczygieł D., Buczny J., Bazińska R. (2012). Emotion regulation and emotional information processing: The moderating effect of emotional awareness. Personal. Individ. Differ..

[111] McKinney C., Morse M., Pastuszak J. (2016). Effective and ineffective parenting: Associations with psychological adjustment in emerging adults. J. Fam. Issues.

[112] Kouros C., Pruitt M., Ekas N., Kiriaki R., Sunderland M. (2017). Helicopter parenting, autonomy support, and students’ mental health and well-being: The moderating role of sex and ethnicity. J. Child Fam. Stud..

[113] Pinquart M. (2017). Associations of parenting dimensions and styles with externalizing problems of children and adolescents: An updated meta-analysis. Dev. Psychol..

[114] Pinquart M. (2017). Associations of parenting dimensions and styles with internalizing symptoms in children and adolescents: A meta-analysis. Marriage Fam. Rev..

[115] Wyatt Kaminski J., Valle L.A., Filene J.H., Boyle C.L. (2008). A meta-analytic review of components associated with parent training program effectiveness. J. Abnorm. Child Psychol..

[116] Turner K.A., Elkins S.R., Walther C.A., Short M.B., Schanding G.T. (2023). Too much of a good thing? Associations among parenting profiles and helicopter parenting. Fam. J..

[117] Parolin L.A.L., Benzi IM A., Fanti E., Milesi A., Cipresso P., Preti E. (2021). Italia Ti Ascolto [Italy, I am listening]: An app-based group psychological intervention during the COVID-19 pandemic. Res. Psychother. Psychopathol. Process Outcome.

