# Supplementary material for: Mentalized Affectivity, Helicopter Parenting, and Psychopathological Risk in Emerging Adults: A Network Analysis
Source: Eur J Investig Health Psychol Educ. 2024 Sep 18;14(9):2523–41. doi: 10.3390/ejihpe14090167 (PMC11431566; doi:10.3390/ejihpe14090167)
Supplement: Supplementary file 1 [file ejihpe-14-00167-s001.zip › ejihpe-3189363-supplementary.pdf]

Supplemental Materials

Data Analytic Strategy for Network estimation and assessment

The data analysis was performed using various R packages, including *qgraph*, *bootnet*, *igraph*, *networktools*, and *tidygraph*. Additionally, the *lavaan*, *corrplot*, *summarytools*, and *psych* packages were utilized for data analysis. Initially, the item pool underwent a meticulous review to ensure data accuracy and identify any missing values. Furthermore, variables' normality assessment was performed. Bootstrap procedures were employed to assess the network's stability and consistency. A total of 1,000 bootstrap samples were generated to estimate confidence intervals for both centrality and bridge indices.

Stability coefficients were calculated for the strength, expected influence centrality indices, and bridge strength. These coefficients indicate the maximum proportion of observations that can be omitted while maintaining a 0.7 correlation between the statistics calculated in the full dataset and those in a subset of observations, with 95% confidence. The optimal value for correlation stability coefficients is around 0.75 (Epskamp et al., 2018).

Subsequently, correlation networks were constructed using the Graphical Least Absolute Shrinkage and Selection Operator (GLASSO) algorithm. Regularized partial correlations were estimated with the Extended Bayesian Information Criterion (EBICglasso), setting the tuning parameter to 0.5 (Foygel & Drton, 2010; Friedman et al., 2008). The Fruchterman-Reingold force-directed algorithm was employed to enhance the visualization of network edges and clustering structures while preventing node overlap (Fruchterman & Reingold, 1991; Jones et al., 2021).

Following this, centrality indices were computed to identify key nodes, including expected influence, strength, closeness, and betweenness. Both raw and standardized centrality scores were analyzed for comprehensive insights, with higher values indicating

greater node centrality. Expected influence is the sum of edge weights between a central node and all its connections within the network. Strength, like expected influence, is the sum of absolute edge weights. In the absence of negative correlations, both indices provide identical results (Bringmann et al., 2019; Costantini et al., 2015).

Closeness centrality measures a node's proximity to all others in the network, calculated as the reciprocal of the sum of shortest path distances from the node to all others (Epskamp et al., 2018). Higher closeness scores indicate nodes that can efficiently disseminate information due to shorter paths, crucial for rapid interaction (Burger et al., 2022). Betweenness centrality quantifies the frequency with which a node acts as a bridge along the shortest path between other nodes (Epskamp et al., 2018; Burger et al., 2022). Nodes with high betweenness centrality exert significant influence by controlling information flow between nodes. Moreover, bridge centrality analysis was performed to identify nodes connecting distinct psychological constructs within the network. This analysis is crucial for pinpointing intervention points to disrupt harmful pathways or reinforce beneficial ones. Metrics such as bridge strength and bridge expected influence, calculated at one-step and two-step distances, highlighted nodes serving as essential connectors across clusters or communities, potentially regulating symptom propagation (Jones, 2017; Jones et al., 2019).

In the final step, network group invariance was assessed using the Network Comparison Test (NCT) to determine the consistency of the network structure across assigned sex at birth, ensuring equivalence between males and females. NCT is a permutation-based statistical technique that compares network structures across different populations (van Borkulo et al., 2023). It evaluates three core aspects: the invariance of network structure, edge strength, and global strength. First, the observed data are analyzed to estimate network structures and calculate the test statistic. Second, a reference distribution is created by pooling the observed data sets, repeatedly resampling them to match the original

sample sizes, estimating the network structures, and calculating the test statistic. This step typically involves a few thousand iterations to ensure accuracy. Third, the significance of the observed test statistic is evaluated by comparing it to the reference distribution, with the p-value indicating the proportion of resampled test statistics that are as extreme as the observed statistic. The NCT is implemented in the R package *NetworkComparisonTest* and is particularly useful for analyzing psychological networks and identifying differences between groups, such as gender differences in symptom networks (Borsboom, 2017; Borsboom & Cramer, 2013; Epskamp et al., 2018).

*Figure 1S. Bootstrapped Confidence Intervals of Estimated Edge Weights for the Network (*N *= 913)*

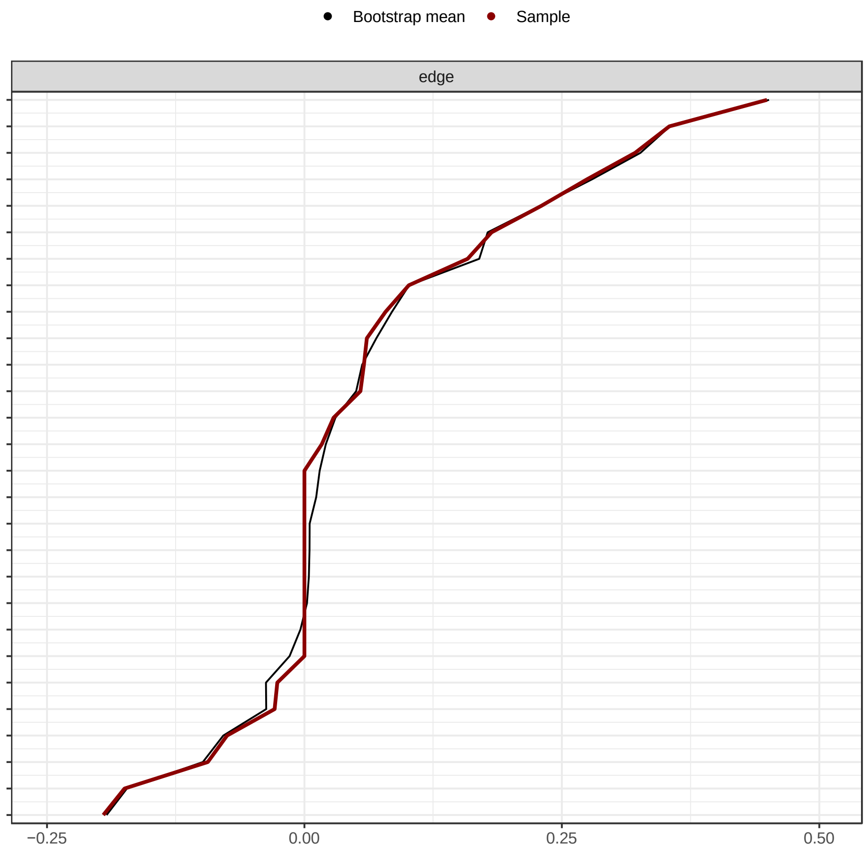

*Figure 2S. Plotted Case Dropping for Strength Centrality Index of the Network (*N *= 913)*

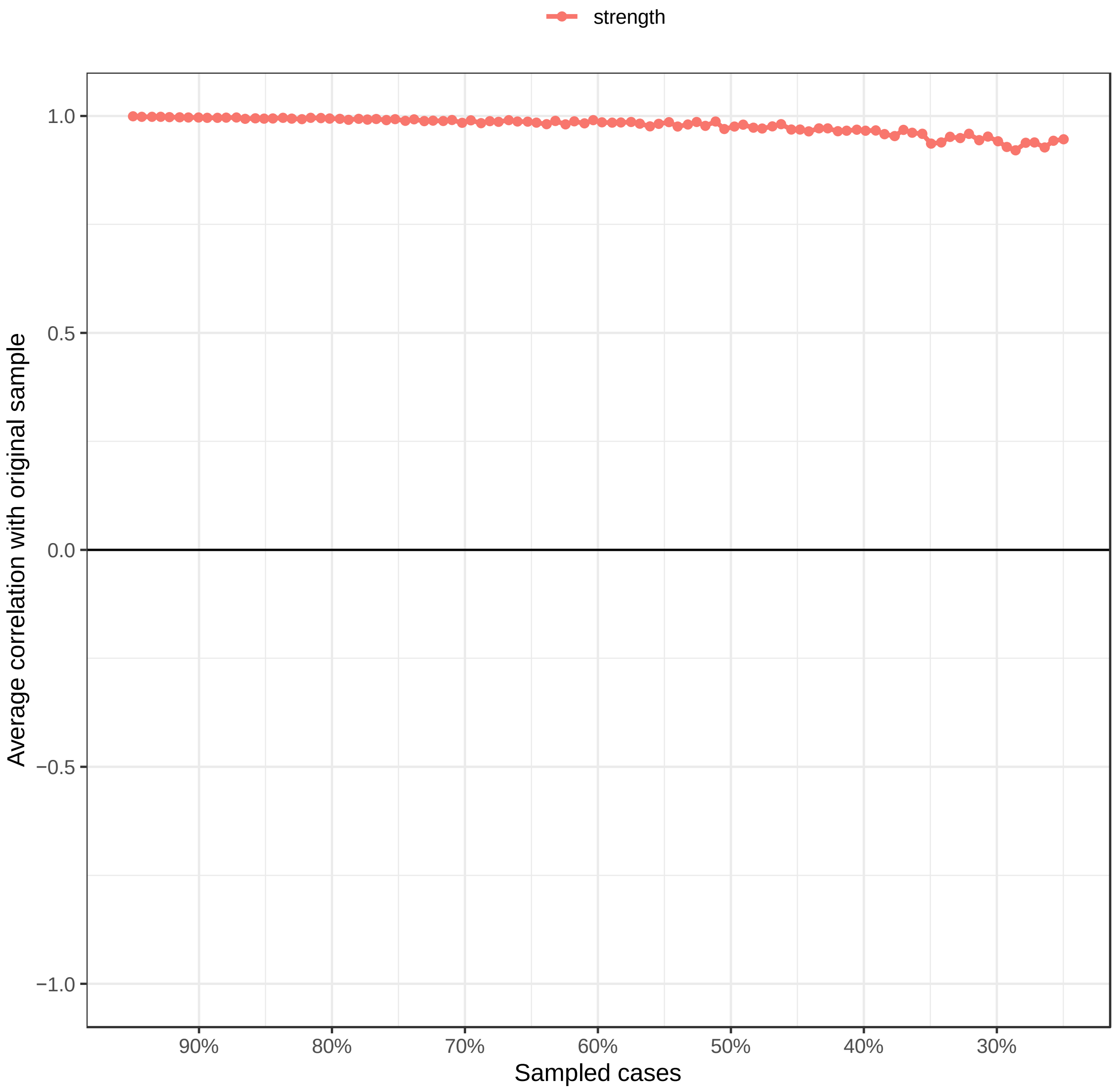

*Figure 3S. Bootstrapped Expected Influence Difference Test of the Network Variables (*N *= 913)*

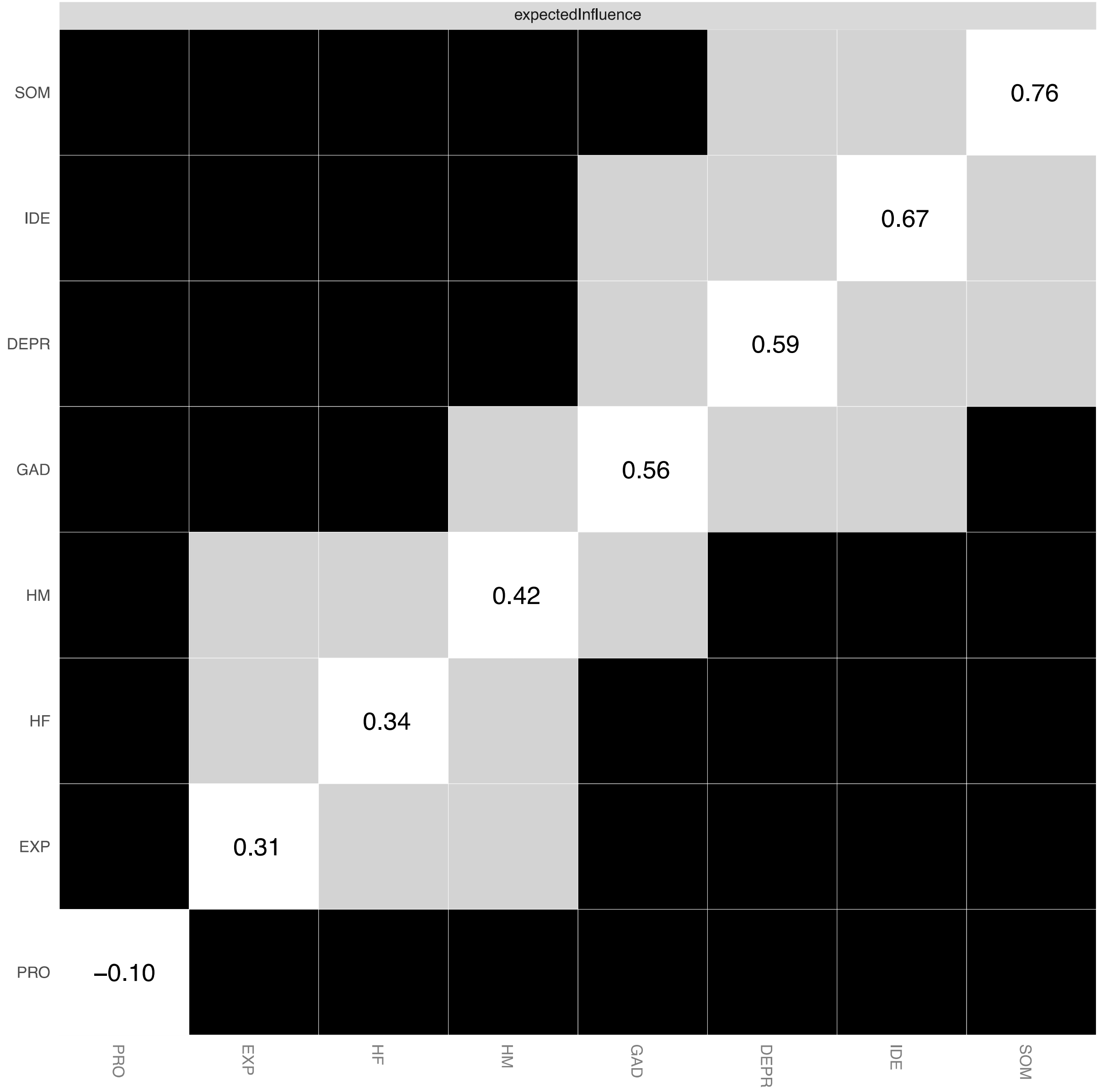

Note. Gray-colored boxes represent non-significant differences between two nodes, and black boxes represent significant differences between two nodes ($p < .05$). DEPR = Depressive symptoms; GAD = Anxiety; SOM = Somatization; HF = Paternal Helicopter Parenting; HM = Maternal Helicopter Parenting; IDE = Identifying Emotions; PRO = Processing Emotions; EXP = Expressing Emotions.

*Figure 4S. Bootstrapped Strength Centrality Index Difference Test of the Network Variables (*N *= 913)*

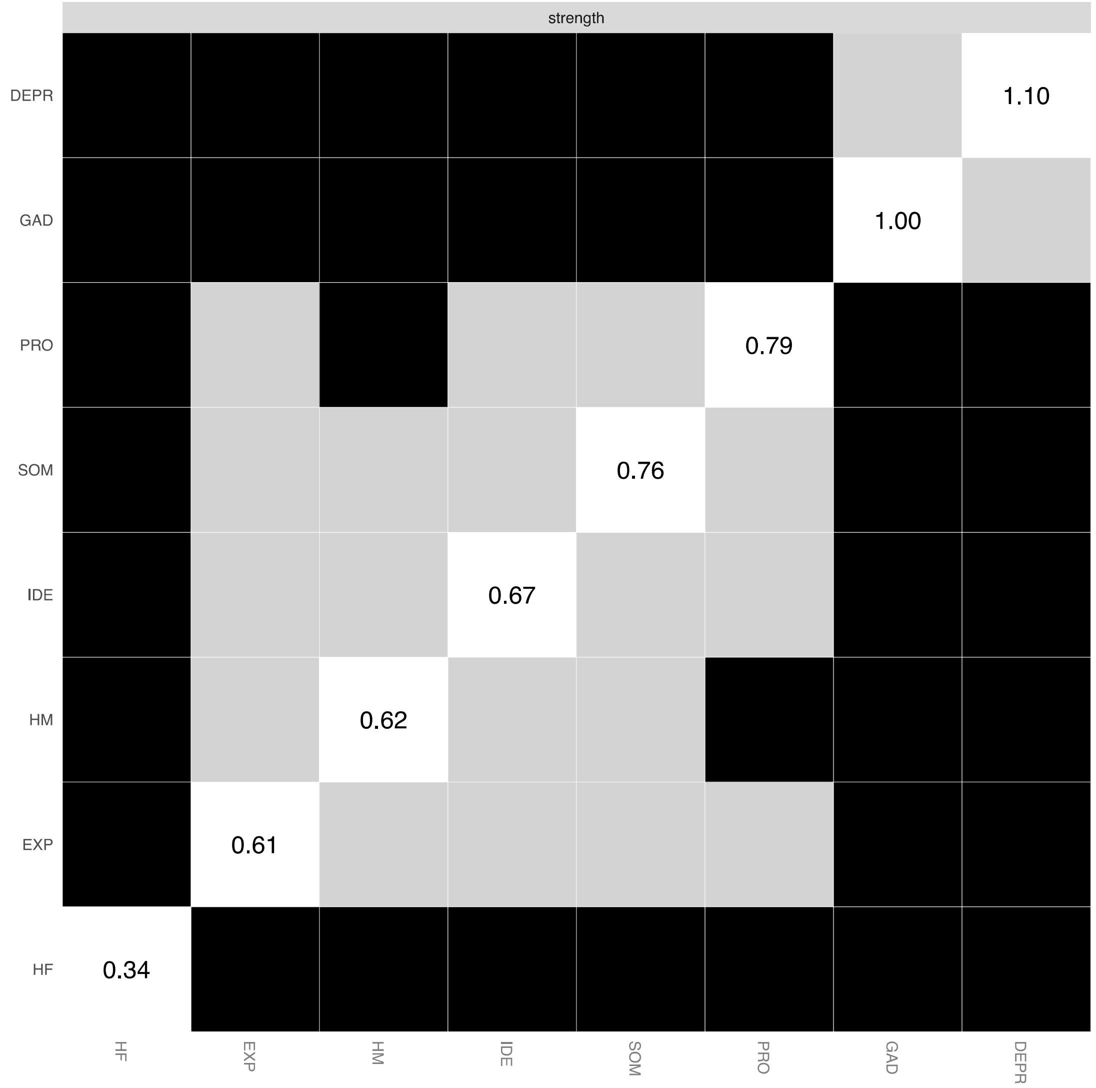

Note. Gray-colored boxes represent non-significant differences between two nodes, and black boxes represent significant differences between two nodes (p < .05). DEPR = Depressive symptoms; GAD = Anxiety; SOM = Somatization; HF = Paternal Helicopter Parenting; HM = Maternal Helicopter Parenting; IDE = Identifying Emotions; PRO = Processing Emotions; EXP = Expressing Emotions.

*Figure 5S. Bridge Centrality Indices of the Network Variables (*N *= 913)*

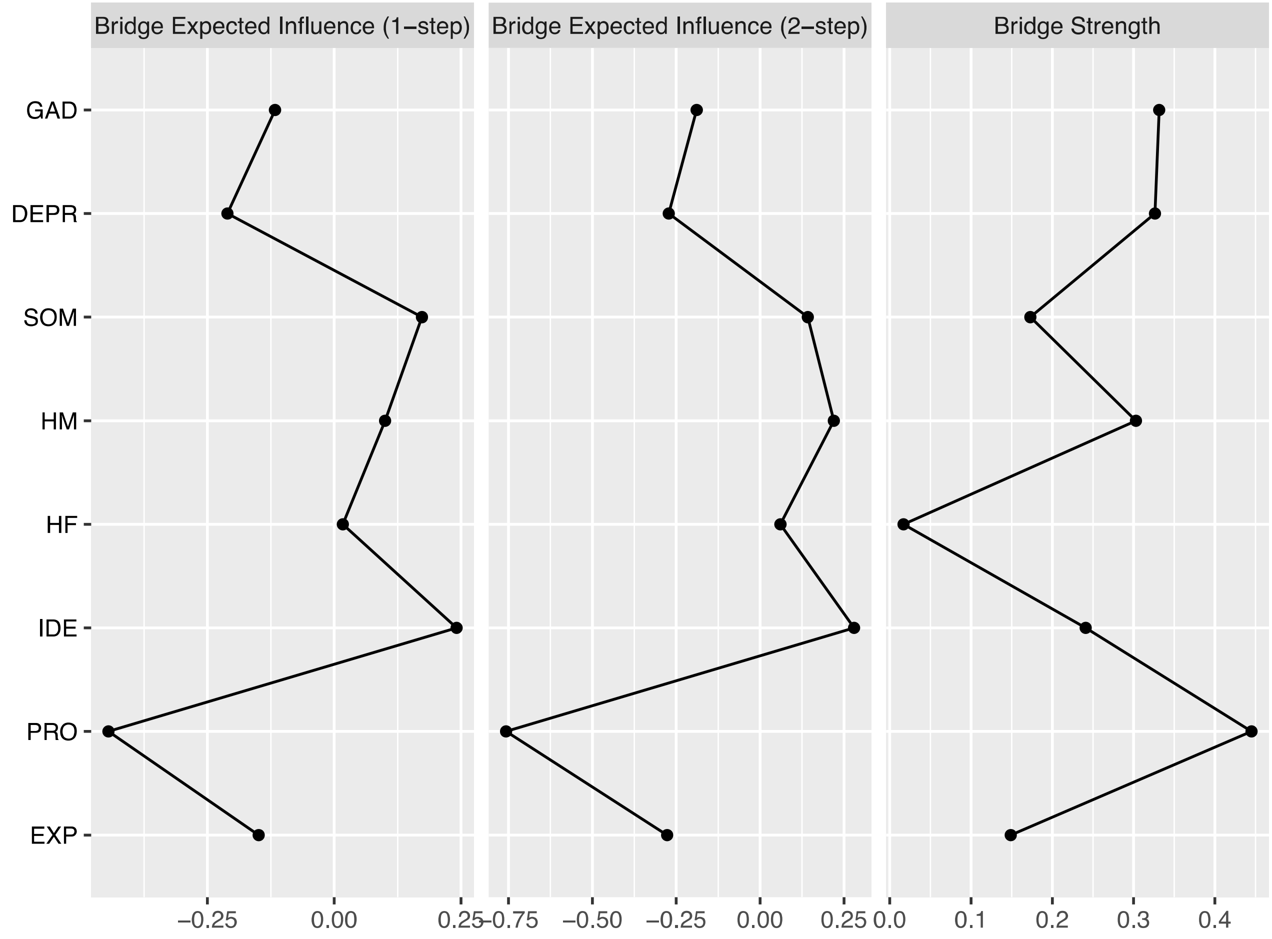

Note. Bridge Centrality indices of the network variables (i.e., Bridge Strength, Bridge Expected Influence (1-step), Bridge Expected Influence (2-step)) are shown as standardized z-scores. DEPR = Depressive symptoms; GAD = Anxiety; SOM = Somatization; HF = Paternal Helicopter Parenting; HM = Maternal Helicopter Parenting; IDE = Identifying Emotions; PRO = Processing Emotions; EXP = Expressing Emotions.

*Figure 6S. Bootstrapped Bridge Strength Difference Test of the Network Variables (*N *= 913)*

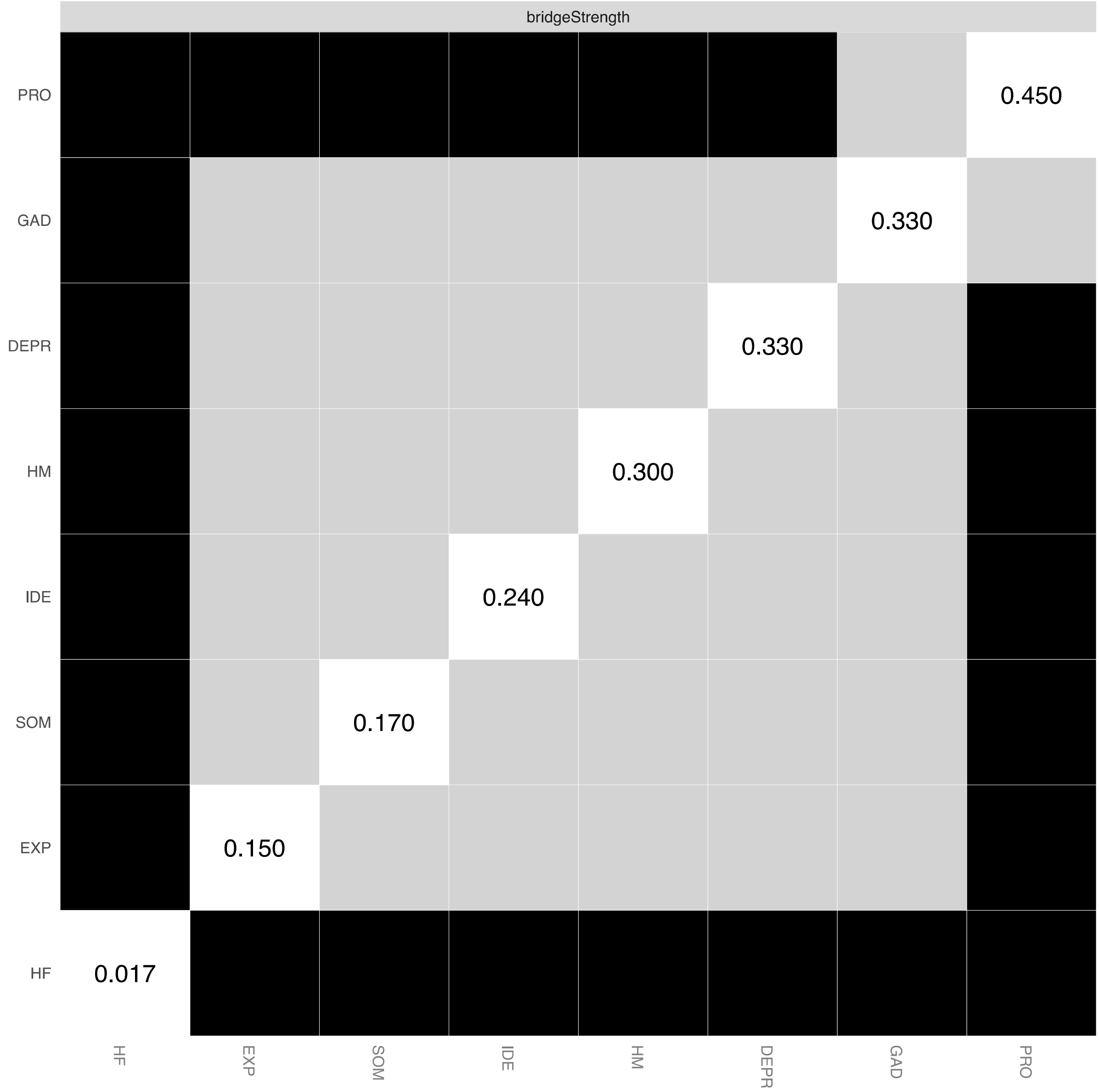

Note. Gray-colored boxes represent non-significant differences between two nodes, and black boxes represent significant differences between two nodes ($p < .05$). DEPR = Depressive symptoms; GAD = Anxiety; SOM = Somatization; HF = Paternal Helicopter Parenting; HM = Maternal Helicopter Parenting; IDE = Identifying Emotions; PRO = Processing Emotions; EXP = Expressing Emotions.

*Figure 7S. Plot of Node Predictability Depicting Psychopathology, Mentalized Affectivity, and Helicopter Parenting Scales in Emerging Adults (*N *= 913)*

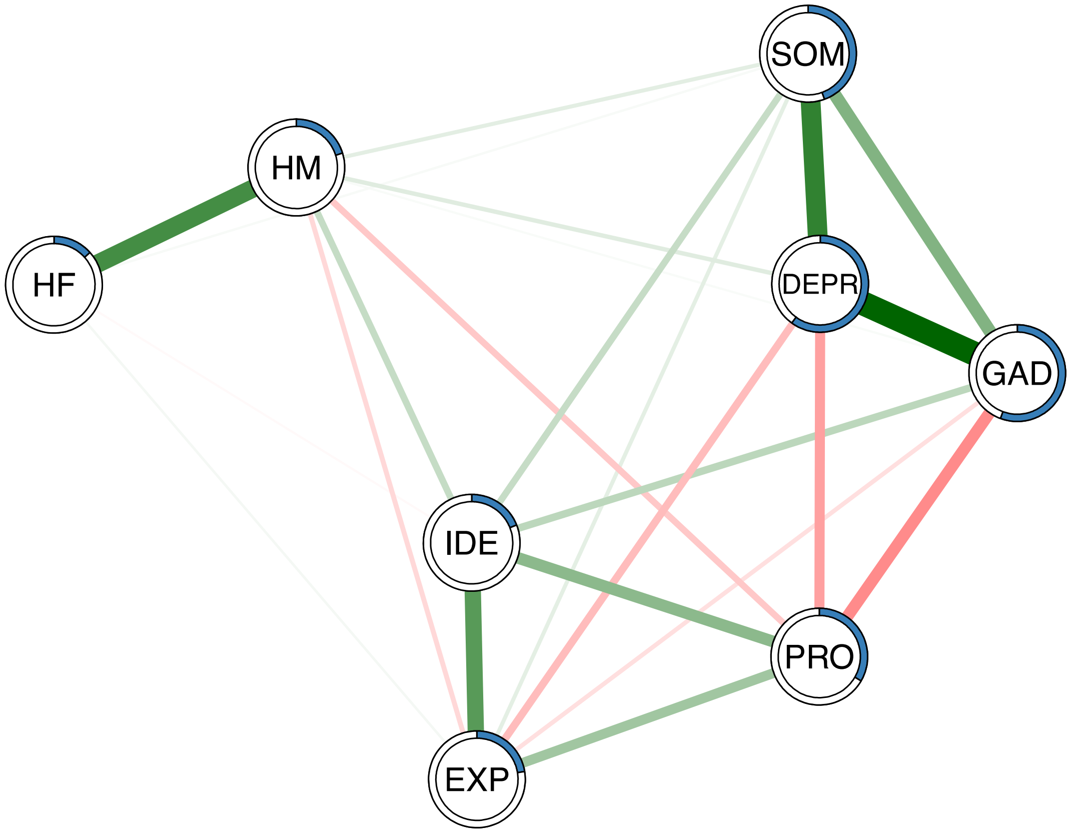

Note. Nodes represent variables, and edges depict the relation between two variables, controlling for all other variables. Thicker edges indicate stronger positive partial correlations. Green edges represent positive associations, and red edges are negative associations. The blue color indicates the R^2 of each node. DEPR = Depressive symptoms (R^2 =.60); GAD = Anxiety (R^2 =.56); SOM = Somatization (R^2 =.44); HF = Paternal Helicopter Parenting (R^2 =.13); HM = Maternal Helicopter Parenting (R^2 =.20); IDE = Identifying Emotions (R^2 =.19); PRO = Processing Emotions (R^2 =.33); EXP = Expressing Emotions (R^2 =.22).

*Figure 8S. Bootstrapped Edge-Weights Difference Test of the Network Variables (*N *= 913)*

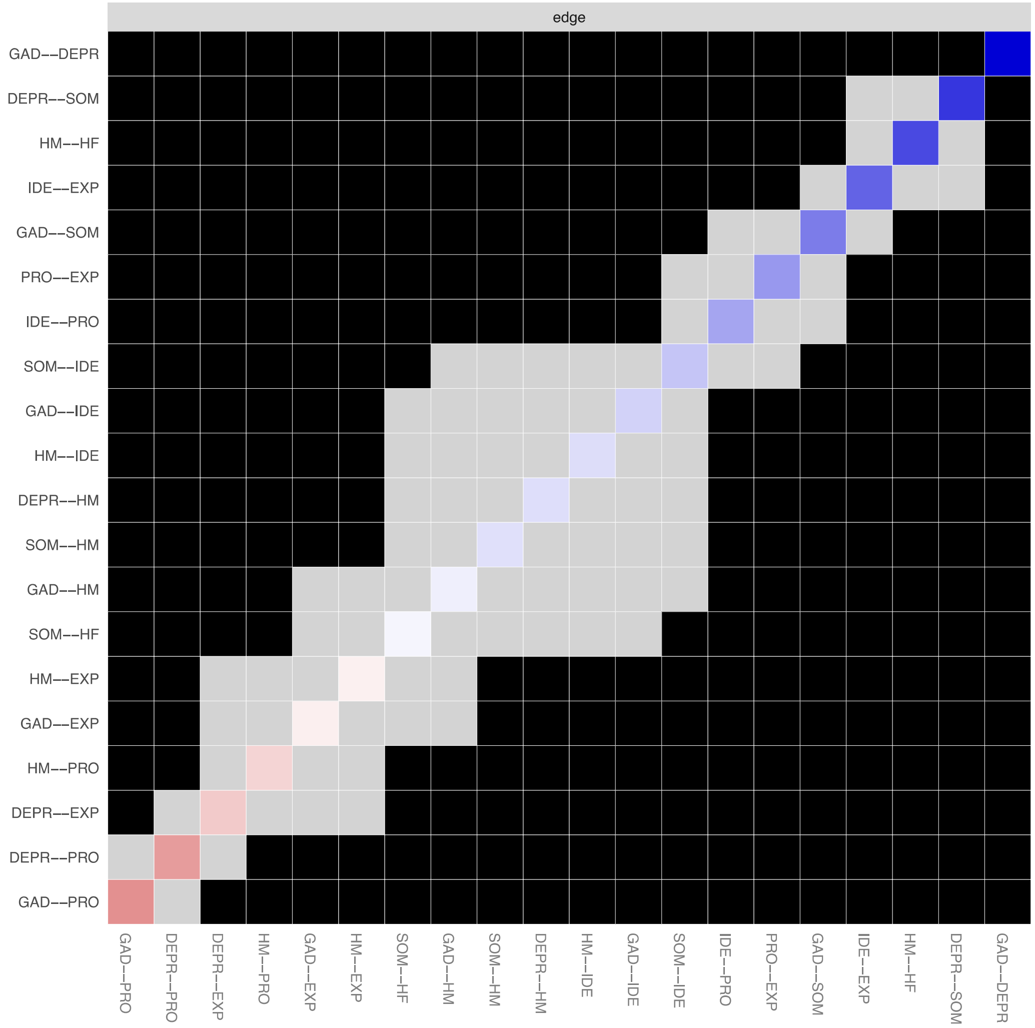

Note. Diagonal indicates the edge colors with the corresponding direction and magnitude of the associations. Gray-colored boxes represent non-significant differences between two nodes, and black boxes represent significant differences between two nodes ($p < .05$). DEPR = Depressive symptoms; GAD = Anxiety; SOM = Somatization; HF = Paternal Helicopter Parenting; HM = Maternal Helicopter Parenting; IDE = Identifying Emotions; PRO = Processing Emotions; EXP = Expressing Emotions.
